# Clinical Evidence on the Use of Propolis for Oral Mucositis

**DOI:** 10.3390/ph19030425

**Published:** 2026-03-05

**Authors:** Matheus de Morais Assis, Barbara Hana Silva Morales Pino, Larissa Kaori Maquedano, Felipe Gustavo Carvalho, Fernando Augusto Lima Marson, Giovanna Barbarini Longato

**Affiliations:** 1 Research Laboratory in Molecular Pharmacology of Bioactive Compounds, Postgraduate Program in Health Sciences, São Francisco University (USF–Universidade São Francisco), Bragança Paulista 12916-900, SP, Brazil; assis.matheus@outlook.com.br (M.d.M.A.); studies.barbara@gmail.com (B.H.S.M.P.); larissamaquedano8@gmail.com (L.K.M.); felipe_gustavo_carvalho@hotmail.com (F.G.C.); 2 Laboratory of Molecular Biology and Genetics, LunGuardian Research Group—Epidemiology of Respiratory and Infectious Diseases, Postgraduate Program of Health Sciences, Postgraduate Program in Health Data Science, São Francisco University (USF–Universidade São Francisco), Bragança Paulista 12916-900, SP, Brazil; fernando.marson@usf.edu.br

**Keywords:** clinical trials, hospital dentistry, oral mucositis, propolis

## Abstract

Cancer represents a major global public health challenge, and its treatments, such as chemotherapy and radiotherapy, are frequently associated with adverse effects. Among these, oral mucositis (OM) stands out as a debilitating inflammatory condition that compromises quality of life and may lead to interruption of cancer therapy. Given the limited efficacy of conventional treatments, propolis has been investigated as a natural therapeutic alternative due to its antioxidant, anti-inflammatory, and antimicrobial properties. This narrative literature review, conducted between 2012 and 2025, included studies indexed in the Public Medical Database (PubMed), Scientific Electronic Library Online (SciELO), Literatura Latino-Americana e do Caribe em Ciências da Saúde (LILACS), Google Scholar, and ClinicalTrials.gov databases. We analyzed clinical trials that evaluated different forms of propolis administration, such as gel, mouthwashes, oral solutions, and topical applications, in patients with various types of cancer undergoing radiotherapy, chemotherapy, or combined treatment. The results demonstrated a significant reduction in pain, dysphagia, dysgeusia, and OM severity, as well as a delay in the onset and progression of lesions, with a low incidence of adverse effects. However, variability in the chemical composition of propolis and the lack of standardized protocols still limit the reproducibility and comparability of the findings. Overall, these results reinforce the therapeutic potential of propolis for the prevention and treatment of OM in oncology patients, while underscoring the need for long-term, randomized clinical trials to establish optimal concentrations, pharmacological formulations, and standardized application protocols.

## 1. Introduction

Cancer is a malignant neoplasm resulting from genomic alterations and disruptions in the regulatory mechanisms of the cell cycle [1]. It represents one of the major global public health challenges [2] and, in Brazil, is the second leading cause of death among individuals under 70 years of age [3]. Multimodal therapeutic strategies, combining transplantation, surgery, chemotherapy, and radiotherapy, remain the cornerstone for the management of primary, recurrent, and metastatic cases [4]. However, these modalities, particularly chemotherapy and radiotherapy, are associated with significant adverse effects, especially in tissues with a high cell turnover, such as the oral epithelium [5].

Mucositis is an inflammatory complication that can affect the entire gastrointestinal tract and is termed oral mucositis (OM) when located in the oral cavity. It is among the most prevalent and debilitating adverse effects of cancer therapy. When induced by chemotherapy, OM typically manifests during the first week of treatment, peaking around the eighth day; in radiotherapy-associated cases, severity usually increases from the second week onward [6]. Clinically, OM is characterized by erythema, ulceration, desquamation, pain, and functional impairment, including difficulty with chewing, swallowing, and speaking. Its pathophysiology, particularly in chemoradiotherapy-induced cases, progresses through five distinct phases: initiation, signaling, amplification, ulceration, and healing, involving cellular injury, an exacerbated inflammatory response, and subsequent tissue regeneration [7].

The World Health Organization (WHO) classifies OM into five grades. Grade 0 corresponds to asymptomatic patients without pain or ulceration; Grade 1 is characterized by erythema and pain without ulceration; Grade 2 involves ulceration and pain, with preserved ability to ingest solid foods; Grade 3 is marked by ulceration and pain that restrict oral intake to liquids; and Grade 4, the most severe form, requires enteral nutritional support, typically via a nasogastric tube [8].

Current management strategies for OM focus on prevention, symptom control, and mitigation of lesion severity. These include mouth rinses containing corticosteroids, chlorhexidine, or anesthetic agents; topical ointments and gels; and well as systemic medications and nutritional supplements [9]. In this context, natural products have gained increasing attention as potential therapeutic alternatives due to their accessibility and broad acceptance. Propolis, a resinous substance produced by bees, exhibits antioxidant, anti-inflammatory, and antimicrobial properties that make it a compelling candidate for the management of OM, particularly in patients undergoing cancer therapy [10].

Although reviews addressing the use of propolis in OM management are available, the present review differs by focusing exclusively on evidence derived from contemporary clinical trials and by providing a structured analysis of the different pharmaceutical formulations in relation to clinical efficacy. This work represents an updated synthesis and aggregation of existing evidence. It is noteworthy that a literature gap exists concerning reviews from the past two years focused on propolis as a therapeutic approach for oral mucositis, which justifies the demand for up-to-date investigations. Therefore, the aim of this review is to critically evaluate the current clinical evidence on the use of propolis for the prevention and treatment of OM. To this end, the therapeutic benefits, routes of administration, safety profiles, methodological limitations, and the pressing need for standardized clinical protocols are highlighted.

## 2. Materials and Methods

This study is a narrative literature review aimed at describing and analyzing the available clinical evidence on the use of propolis for the prevention and treatment of OM in oncology patients. The bibliographic search was conducted between January 2024 and September 2025 and included publications from January 2012 to early 2025.

The following electronic databases were searched: PubMed (MEDLINE), Scientific Electronic Library Online (SciELO), ClinicalTrials.gov, and Google Scholar. Only original clinical trials published in English, Spanish and Portuguese were included. Due to the high volume of unfiltred results and the potential for non-relevant records in Google Scholar, screening in this database was limited to the first 10 pages of results sorted by relevance.

**Figure 1:**
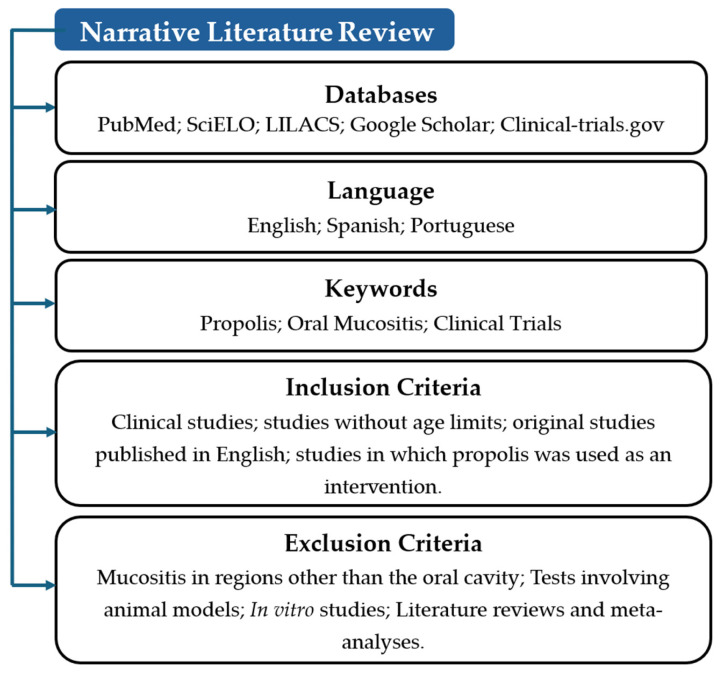
Flowchart illustrating the identification, screening, eligibility assessment, and final inclusion of studies in the narrative review. The diagram summarizes database searches, duplicate removal, title and abstract screening, full-text evaluation, and the final number of clinical trials included.

Controlled vocabulary terms (MeSH and DeCS) were combined with free-text keywords using Boolean operators (AND, OR). The primary search strategy was: (“propolis” OR “bee glue”) AND “oral mucositis” AND “clinical trial”. References retrieved from multiple databases were managed using Microsoft Excel to identify and remove duplicate records prior to screening (Figure 1).

The inclusion criteria were: (1) original clinical trials; (2) studies involving patients of any age with a cancer diagnosis undergoing chemotherapy, radiotherapy, or combined treatment modalities; (3) studies evaluating propolis, in any pharmaceutical formulation, as a therapeutic intervention or adjuvant; and (4) studies in which OM was assessed as an outcome.

Exclusion criteria comprised: (1) in vitro or in vivo animal studies; (2) studies addressing mucositis in anatomical sites other than the oral cavity; (3) systematic reviews, narrative reviews, meta-analyses, editorials, letters to the editor, and case reports; (4) duplicate publications; and (5) studies with insufficient data or incomplete reporting that precluded adequate data extraction.

The literature selection was based on a comprehensive search of titles and abstracts to identify studies relevant to the review’s scope. Full-text assessments were conducted by two reviewers to ensure the quality and pertinence of the included data. The selection process was performed collaboratively to ensure a broad and balanced overview of the current evidence, focusing on the most significant advancements in the field. Data extraction focused on the following variables: authorship and year of publication, country, study design, sample size, oncological treatment protocol, propolis formulation, dosage regimen, and main clinical outcomes. Methodological aspects, including randomization procedures, presence of control groups, blinding, and sample size limitations, were critically appraised to determine the overall strength of the evidence. No restrictions were applied regarding minimum sample size or the geographic origin of propolis, allowing the inclusion of pilot studies, emerging research involving propolis from diverse sources, and novel pharmaceutical formulations in early phases of clinical investigation.

## 3. Results

### 3.1. Characteristics of the Studies

Sixteen studies published between 2012 and 2025 were included in this review (Table 1); half of them (n = 8; 50%) [11,12,13,14,15,16,17,18] were published after 2018. Collectively, these studies encompassed a total of 925 participants.

Regarding the methodological design, the majority of studies (n = 13; 81.25%) were classified as randomized clinical trials. Among these, 7 (43.75%) demonstrated high methodological rigor, employing double- or triple-blind designs [11,14,19,20,21,22,23], whereas 6 (37.5%) were conducted as open-label or single-blind trials [12,13,15,18,24,25]. In addition, 1 (6.25%) prospective non-randomized clinical trial was included [17], along with 2 (12.5%) uncontrolled phase II studies [16,26].

In terms of geographical distribution, 5 (31.3%) studies were conducted in the Middle East [11,15,20,21,22], 5 (31.3%) in Europe [16,17,19,23,25], 2 (12.5%) in South America [14,26], 3 (18.8%) in East Asia [12,13,18], and 1 (6.3%) in Africa [24]. Notably, Italy and Iran each contributed three studies, while Brazil contributed two.

With respect to the form of intervention, 13 (81.3%) studies evaluated topical administration [11,12,13,14,15,16,17,18,19,21,22,23,24,26]. Two (12.5%) studies combined topical and systemic administration, consisting of oral rinsing followed by swallowing [11,20], and 1 (6.3%) study investigated exclusive systemic administration via oral tablets [25]. Among the topical formulations, mouthwash was the most frequently used (46.2%) [13,14,18,21,24], followed by moisturizing gel (15.4%) [12,17], preparations combined with other bioactive compounds (15.4%) [23,24], mucoadhesive gel (7.7%) [26], ethanolic extract (7.7%) [19], and aqueous solution (7.7%) [11].

**Table 1:** Summary of the characteristics of clinical studies evaluating the potential of propolis in the prevention and treatment of oral mucositis.

Author (Ref.)	Year	Type of Study	Country	N	Product and Method of Administration	Main Results	Conclusions
Abdulrahman [24]	2012	Phase II randomized controlled clinical trial, unblinded	Egypt	90	Blend of honey, olive oil, propolis extract, and beeswax (HOPE; topical application)	The severity (grade) of oral mucositis (OM) did not differ between groups; however, HOPE promoted faster healing in patients with chemotherapy-induced grade 3 OM	The authors suggested the inclusion of honey, other bee-derived products, and olive oil in future therapeutic trials for chemotherapy-induced OM
Tomazevic [19]	2013	Randomized, double-blind, placebo-controlled study	Slovenia	40	70% ethanolic propolis extract (topical application)	Severe OM showed a slightly shorter duration and reduced extent in the propolis group; however, differences in frequency, mean duration, and mean severity were not statistically significant	The authors did not recommend propolis for the treatment of severe OM
Noronha [26]	2014	Phase II clinical study	Brazil	24	5% propolis mucoadhesive gel (topical application)	Twenty patients did not develop OM; two developed grade 1 and two developed grade 2 OM	Propolis mucoadhesive gel may represent a useful topical option for the prevention of radiation-induced OM
Bolouri [20]	2015	Triple-blind, randomized, placebo-controlled clinical trial	Iran	20	3% aqueous propolis solution used as rinse, gargle, and swallow (topical and systemic exposure)	OM scores were lower in the propolis group; eight patients showed no evidence of OM throughout radiotherapy	Propolis-based mouthwash appeared to be a safe and effective option for preventing and treating radiotherapy-induced OM
Eslami [22]	2016	Double-blind randomized clinical trial	Iran	72	Propolis mouthwash (topical application)	Clinical examination at days 5 and 10 showed that 50% of patients in the propolis group were OM-free, and none developed grade 4 OM	Propolis mouthwash was associated with a reduction in chemotherapy-related oral complications
Karbassi [21]	2016	Randomized, double-blind, placebo-controlled clinical trial	Iran	40	30% propolis mouthwash (topical application)	Improvements were observed in erythema, ulceration, ability to eat and drink, and overall OM severity in the propolis group	Oral care with propolis mouthwash was an effective intervention for improving oral health during chemotherapy
Marucci [23]	2016	Double-blind randomized phase III study	Italy	104	Faringel^®^—a formulation containing 6% propolis, aloe vera, calendula, and chamomile (topical application)	A total of 61 patients developed grade 3 OM, with no significant differences in OM severity between groups	The formulation containing propolis and other natural ingredients was not effective in preventing acute OM
Piredda [25]	2017	Randomized controlled pilot trial	Italy	60	Tablets containing 8% dry propolis extract (systemic administration)	No patient in the experimental group developed OM above grade 1 during the first treatment cycle	Propolis tablets combined with sodium bicarbonate mouthwash were safe, well tolerated, and showed promising results in OM prevention
Dastan [11]	2020	Randomized, double-blind clinical trial	Iran	30	Propolis oral solution (0.8 mg/mL) for rinsing and swallowing (topical and systemic exposure)	No differences were observed in the first week; however, in weeks 3 and 4, grade 3 OM occurred in 0% of the propolis group versus 33.33% and 13.33% in the placebo group, respectively	Propolis mouthwash was effective and safe in reducing OM severity and dysphagia during head and neck radiotherapy
Nakao [12]	2021	Randomized controlled pilot trial	Japan	25	Propolis oral moisturizing gel (topical application)	Salivary levels of *Porphyromonas gingivalis* decreased after propolis treatment, and oral pain was relieved in all symptomatic participants	Propolis gel may be a beneficial adjunct in the management of OM during head and neck radiotherapy
Hamzah [13]	2022	Randomized controlled trial	Malaysia	17	2.5% propolis mouthwash (topical application)	Significant differences in OM severity were observed between propolis and placebo groups at all assessment points	The 2.5% propolis mouthwash was safe and effective in reducing OM severity
Fernandes [14]	2022	Double-blind randomized clinical trial	Brazil	60	0.8% aqueous propolis suspension (topical application)	Lower mean scores for OM, dysphagia, dysgeusia, oral candidiasis, and inflammatory markers (IL-1β and TNF-α) were observed in the propolis group	Topical propolis appeared to be a useful complementary approach for preventing and treating acute oral toxicities induced by radiotherapy
Çakmak [15]	2023	Randomized controlled clinical trial	Turkey	64	Aqueous propolis extract (topical application)	The incidence, duration, and onset of OM—particularly grades 2–3—were significantly reduced in the intervention group	Propolis mouthwash combined with standard oral hygiene delayed OM onset and reduced its incidence and duration
Szabó [17]	2024	Prospective clinical trial	Hungary	127	Nano-bio-fusion (NBF) gingival gel (topical application)	The NBF and pilocarpine group showed improved healing of mucositis associated with xerostomia, especially in severe cases with visible lesions.	NBF gel was beneficial in accelerating OM healing
Jen [18]	2025	Randomized, single-blind, clinical trial with three parallel groups	Taiwan	75	Mouthwash containing 10 g of Thai propolis in 20 mL distilled water (topical and systemic exposure)	Propolis reduced OM severity, particularly between weeks 4 and 11; severe OM (WHO ≥ 3) was moderately less frequent	Although propolis reduced OM severity, the lack of significant reduction in incidence highlights the need for further optimization studies
Piredda [16]	2025	Single-center, prospective, open-label, uncontrolled phase II study	Italy	77	Faringel^®^—a formulation containing 6% propolis, aloe vera, calendula, and chamomile (topical application)	Oral disease severity decreased significantly, with reductions in oropharyngeal pain and dysgeusia; OM severity decreased by at least one grade or remained absent	The protocol was safe, well tolerated, and effective for managing oral diseases in adult palliative care patients

Abbreviations: IL-1β, Interleukin-1 beta; N, number of participants; OM, oral mucositis; TNF-α, Tumor Necrosis Factor alpha; WHO, World Health Organization.

### 3.2. Participant Selection Criteria

The sixteen included studies comprised a total of 925 participants. Sample sizes varied substantially across trials, ranging from small pilot studies with 17 to 20 participants [13,20] to larger prospective studies enrolling more than 100 patients [17,23]. With respect to age distribution, the vast majority of studies focused on adult populations. Nevertheless, two clinical trials specifically evaluated pediatric cohorts: one involving children and adolescents aged 2 to 18 years with acute lymphoblastic leukemia [24], and another including patients aged 1 to 19 years undergoing chemotherapy [19].

From a clinical perspective, the studies can be broadly stratified into two major groups based on tumor location and type of antineoplastic treatment. The most extensively investigated subgroup consisted of patients with head and neck cancer undergoing radiotherapy or chemoradiotherapy [2,4,11,14,18,20,21,23,26]. These participants typically presented with carcinomas involving the nasopharynx [13], oral cavity, tongue, hypopharynx, larynx, salivary glands, or paranasal sinuses [11,18]. Radiotherapy protocols generally involved curative doses ranging from 40 Gy [14] to 70 Gy [12,20], most commonly delivered using intensity-modulated radiotherapy or three-dimensional conformal radiotherapy [14].

A second major group comprised patients receiving cytotoxic chemotherapy for systemic malignancies. This group included individuals with breast cancer treated with doxorubicin and cyclophosphamide [23], patients with acute lymphoblastic leukemia [5,22,24], and those undergoing hematopoietic stem cell transplantation [15]. In addition, more recent studies expanded inclusion criteria to encompass distinct clinical scenarios, such as patients receiving palliative care with limited life expectancy [16] and individuals with persistent xerostomia secondary to antineoplastic therapy, hormonal disorders, or Sjögren’s syndrome [17]. In several trials, additional eligibility requirements included a minimum age of 18 years, absence of enteral nutrition via nasogastric tube or gastrostomy, and adequate bone marrow, hepatic, and renal function [23].

Regarding eligibility criteria, most studies applied strict exclusion criteria to minimize potential confounding factors. Participants were frequently excluded if they had a known hypersensitivity to bee-derived products (e.g., propolis or honey) or other components of the study formulations [6,13,14,18,19,22,25,26]. Other common exclusion criteria included active oral infections, such as advanced periodontal disease (periodontal pockets ≥ 6 mm) [24,25] or poor dentition [25], as well as severe systemic comorbidities, including uncontrolled diabetes mellitus [5,18,22,24], hypertension, autoimmune diseases [5], renal or hepatic insufficiency [5,11], and mental disorders [18]. Furthermore, recent use of antibiotics, chronic corticosteroids, anticoagulants, or other investigational drugs within the preceding 30 days constituted grounds for exclusion in several protocols [11,23,25]. Patients unable to swallow study formulations or unwilling to discontinue tobacco and alcohol consumption were also commonly deemed ineligible [16,26].

### 3.3. Pharmaceutical Formulations, Chemical Composition, and Dosing Protocols

The included studies exhibited substantial heterogeneity with respect to pharmaceutical formulations, chemical composition, and administration protocols of propolis. Concerning the delivery route, the majority of trials (n = 13; 81.25%) employed topical administration directly to the oral mucosa. Among these, mouthwashes represented the most commonly used formulation, being evaluated in seven studies [11,13,15,18,20,21,22]. Propolis concentrations varied widely, ranging from 0.08% (0.8 mg/mL) [11] to 30% [21], including intermediate dilutions of 2.5% [13]. The administered volumes per application ranged from 5 mL [21] to 20 mL [11], with intermediate volumes of 7 mL [13,16], 10 mL [18,22], and 15 mL [20].

Gel-based formulations were investigated in four studies, with distinct dosing regimens and patient-reported tolerability profiles. A 5% mucoadhesive gel demonstrated good patient acceptability when applied every eight hours [26]. In contrast, a moisturizing gel formulation was reported to have an unpleasant viscosity by a minority of participants; nevertheless, a nighttime application of 1 mL was considered effective [12]. In addition, a nanoemulsion gingival gel containing propolis, vitamin C, and vitamin E was evaluated with a regimen of three daily applications [17].

Three studies assessed compound or multi-herbal formulations. These included Faringel, a preparation combining propolis, Aloe vera, Calendula officinalis, and Matricaria chamomilla, administered in 7 mL doses four times daily [16,23], and HOPE, a formulation consisting of honey, olive oil, propolis extract, and beeswax, applied topically three times per day [24]. Exclusive systemic administration was investigated in only one pilot study, which evaluated oral tablets containing 80 mg of dry propolis extract standardized to galangin, administered in combination with bicarbonate mouth rinses [25].

A critical methodological source of heterogeneity concerned the extraction solvent, which has a significant impact on mucosal tolerability. Most recent trials used aqueous extracts or alcohol-free formulations to minimize local irritation [11,13,14,15,20]. In contrast, one pediatric study employed a 70% ethanolic extract applied directly to oral ulcers; this approach was associated with poor adherence and adverse effects, including vomiting, attributed to the strong taste and pungency of the formulation [19].

With respect to geographical origin and biological type, key determinants for chemical standardization, only a limited number of studies provided detailed characterization of propolis. Identified types included Brazilian green propolis, used in three studies [14,24,26]; Taiwanese green propolis, evaluated in one trial [18]; and Chinese poplar-type propolis, assessed in one study [19]. The remaining studies did not report the botanical origin or chemical profile of the propolis used, thereby limiting reproducibility and cross-study comparability.

Dosing protocols also varied considerably across studies. Some protocols instructed participants to “swish and spit” [13,21], whereas others recommended a “swish and swallow” approach to achieve both local and systemic effects [11,18,20]. Administration frequencies ranged from three to six times daily, and treatment durations varied from short courses of 10 days [24] to continuous use throughout the entire course of radiotherapy [14,23].

### 3.4. Potential Preventive Effect of Propolis on Oral Mucositis

The preventive efficacy of propolis has been demonstrated by its capacity to maintain a subset of patients free from clinical manifestations of OM or to delay the onset of mucosal lesions. In a phase II study involving patients with head and neck cancer, 83.3% of participants treated with a 5% mucoadhesive propolis gel did not develop any signs of OM throughout the entire course of radiotherapy [26]. Similarly, in another randomized trial, 40% of patients using a propolis mouth rinse remained completely free of mucositis, whereas all patients in the placebo group developed some degree of OM [20]. With respect to delayed onset, a statistically significant postponement in the development of grade 2 and 3 OM was observed in the intervention group compared with controls [15]. This protective effect was further corroborated in patients with acute lymphoblastic leukemia, in whom propolis administration significantly reduced the incidence of mucositis during the initial weeks of chemotherapy [22].

Beyond preventing onset, propolis was also effective in limiting the progression of OM to severe stages (WHO grade ≥ 3), which are clinically relevant because they frequently impair oral intake and may necessitate treatment interruption [13,14,25]. In a randomized pilot-controlled study involving patients with breast cancer, none of the patients receiving propolis developed OM above grade 1 during the first chemotherapy cycle, compared with 16.7% of patients in the control group [25]. Consistent findings were reported among patients undergoing head and neck radiotherapy, whereby in the fourth week of treatment, no patients in the propolis group exhibited grade 3 OM, in contrast to 13.3% in the placebo group [11]. In addition, mean mucositis severity scores were consistently lower in the propolis group than in the placebo group during weeks 2, 4, and 6 of treatment [13].

Nevertheless, the available evidence is not entirely consistent. A phase III randomized clinical trial failed to demonstrate a statistically significant preventive effect of propolis or a reduction in progression to advanced stages of OM, highlighting the heterogeneity of findings and the need for further high-quality, standardized studies [23].

### 3.5. Therapeutic Potential of Propolis in Oral Mucositis Associated with Cancer Treatment

Most included studies reported that propolis-based formulations were safe and generally well tolerated by patients undergoing cancer treatment. In controlled clinical trials, no serious adverse events were attributed to propolis use [11,13,20,21].

Several studies evaluated the therapeutic efficacy of propolis in the management of OM induced by radiotherapy and chemotherapy, particularly among patients with head and neck cancer, nasopharyngeal carcinoma, breast cancer, and leukemia [22].

In the context of radiotherapy-induced OM, propolis demonstrated both efficacy and safety, providing analgesic benefits, reducing dysphagia and dysgeusia, and attenuating mucositis severity during critical phases of treatment [11,12,14,18,20]. Among patients with nasopharyngeal carcinoma, propolis was also effective in alleviating OM-related symptoms throughout the course of radiotherapy [13].

Regarding chemotherapy-associated OM, propolis was evaluated in combination with chamomile, *Aloe vera*, and *Calendula officinalis*. Although this multi-herbal formulation did not prevent the development of grade 3 mucositis in patients with head and neck cancer [23], other studies reported beneficial effects in different oncological settings. In patients with breast cancer receiving doxorubicin and cyclophosphamide, propolis significantly reduced OM symptoms; however, a small proportion of participants experienced mild cutaneous reactions following its use [25].

In patients with leukemia, propolis showed favorable therapeutic outcomes, contributing to symptom relief and improved tolerability. Furthermore, the combined use of propolis and honey was associated with accelerated healing of mucosal lesions in cases of acute lymphoblastic leukemia [22,24]. In addition, among patients receiving palliative care, propolis was found to be safe and effective for OM management, leading to significant reductions in odynophagia and dysgeusia [16].

Overall, these findings support the therapeutic potential of propolis as an adjuvant strategy in the management of OM, helping to mitigate the adverse mucosal effects associated with radiotherapy and chemotherapy. Nevertheless, tolerability issues were noted in specific populations. In pediatric patients undergoing chemotherapy, mild intolerance to propolis was reported, characterized by episodes of vomiting in four participants, attributed to the unpleasant taste of the formulation rather than to allergic reactions. In this study, the intervention group exhibited slightly delayed onset and reduced severity of OM compared with controls; however, although clinically relevant, these differences did not reach statistical significance [19].

### 3.6. Laboratory Evaluation of the Anti-Inflammatory and Antimicrobial Effects of Propolis in Oral Mucositis

In addition to clinical endpoints, several studies incorporated laboratory analyses to elucidate the biological mechanisms underlying the effects of propolis in the pathophysiology of OM. The anti-inflammatory properties of propolis were supported by cytokine profiling, which demonstrated significantly lower levels of interleukin-1β (IL-1β) and tumor necrosis factor alpha (TNF-α) in intervention groups compared with control groups [14].

In parallel, one study specifically investigated the antimicrobial activity of a propolis-based moisturizing gel using real-time polymerase chain reaction (RT-PCR) analysis of saliva samples. Although the intervention did not result in a reduction in total oral bacterial load, it significantly inhibited the proliferation of *Porphyromonas gingivalis* and *methicillin-resistant Staphylococcus aureus* (MRSA), both clinically relevant pathogens within the oral microbiota [12].

Collectively, these immunological and microbiological findings provide biological plausibility for the favorable clinical outcomes observed across several trials. They support the hypothesis that propolis exerts its therapeutic effects, at least in part, by modulating the inflammatory amplification phase of mucositis while selectively influencing pathogenic components of the oral microbiome.

### 3.7. Inconsistent or Negative Findings and Adverse Events

Overall, the included studies reported only mild adverse events associated with the physicochemical properties of propolis. In a pediatric study, the use of a 70% ethanolic extract resulted in vomiting in one participant, attributed to its strong taste and mucosal irritation, which led to treatment discontinuation [19]. In other trials, participant withdrawal occurred due to nausea [11,23]. A transient burning sensation following application was reported by 26.7% of patients using the HOPE formulation [24] and by several participants treated with a mucoadhesive gel; notably, this sensation was typically followed by immediate symptomatic relief [26]. Mild cutaneous reactions, which resolved spontaneously after discontinuation of propolis, were observed in 6.7% of patients in one study [25].

Although not consistently classified as adverse events, several studies reported complaints related to the organoleptic properties of propolis-based formulations, including unpleasant taste and texture [18,23], as well as dissatisfaction with the viscosity of gel-based compounds [12]. These factors are clinically relevant, as they may negatively influence treatment tolerability and patient adherence, particularly in pediatric populations [19].

With respect to methodological limitations, one of the main challenges identified was the difficulty in achieving adequate blinding, owing to the characteristic aroma and taste of propolis. In several trials, participants were reportedly able to recognize their allocation group, thereby compromising blinding and increasing the risk of performance and detection bias [18,25]. Additional limitations included small sample sizes [13,20], short follow-up periods (as brief as 7 days) [21], and substantial heterogeneity in study design.

Furthermore, variability in chemical standardization, differences in extraction solvents, and the frequent lack of detailed botanical origin or chemical characterization of propolis [11,13,21,23] limited cross-study comparability and reproducibility. The use of compound formulations combining propolis with additional bioactive substances beyond the extraction solvent [16,17,23,24] further confounded the attribution of observed effects specifically to propolis. The lack of normalized data across different clinical protocols limits the ability to draw definitive clinical inferences and underscores the need for standardized reporting in future research. Collectively, these limitations underscore the need for well-designed, adequately powered randomized trials using standardized propolis formulations and robust blinding strategies.

## 4. Discussion

Oral mucositis (OM) has been extensively investigated over the past three decades; however, despite substantial scientific advances, a universally standardized management strategy capable of ensuring fully effective prevention and treatment has not yet been established. The incidence of this acute complication among oncology patients remains high, affecting approximately 30–40% of individuals receiving conventional chemotherapy, 60–85% of patients undergoing hematopoietic stem cell transplantation, and up to 90% of patients with head and neck cancer treated with radiotherapy or combined chemoradiotherapy. These high rates translate into painful ulcerative lesions, increased susceptibility to secondary infections, compromised oral intake, and frequent interruptions or dose reductions in antineoplastic treatment, ultimately impacting clinical outcomes and quality of life [27].

Current clinical guidelines from the Multinational Association of Supportive Care in Cancer and the International Society of Oral Oncology (MASCC/ISOO) emphasize that basic oral care represents the cornerstone of OM prevention. This includes meticulous daily oral hygiene, effective mechanical plaque control combined with appropriate oral hygiene agents, and continuous oral lubrication, all of which contribute to reducing oral microbial load, preventing secondary infections, and improving patient comfort. The routine use of chlorhexidine is discouraged, as it has not demonstrated preventive or therapeutic efficacy for OM, except when its use is clinically justified by the presence of an active oral infection. At present, benzydamine hydrochloride remains the only pharmacological agent formally recommended for both the prevention and treatment of OM in specific clinical settings [28].

In parallel, photobiomodulation therapy using low-level, non-ionizing laser irradiation has become established as a gold-standard intervention for OM management. This modality activates endogenous chromophores and initiates photophysical and photochemical processes across multiple biological pathways, resulting in enhanced wound healing, tissue regeneration, modulation of immune responses, and attenuation of inflammation and pain. Robust clinical evidence supports its efficacy and safety across different oncological contexts, reinforcing its position in current guidelines [28].

Cryotherapy has also been widely recommended, particularly for patients receiving certain chemotherapy regimens. Its mechanism of action involves vasoconstriction of superficial oral blood vessels, thereby reducing local exposure of the oral mucosa to circulating cytotoxic agents and limiting tissue damage. Additional therapeutic approaches have been proposed, including the use of anti-inflammatory and analgesic agents. For example, 0.2% morphine mouth rinses have demonstrated significant analgesic effects and improvements in patient-reported quality of life. Nevertheless, for many other pharmacological and non-pharmacological interventions, the available evidence remains insufficient to support formal guideline recommendations [28].

Given the limitations of conventional symptomatic therapies and the growing interest in safer bioactive compounds, propolis has emerged as a potentially relevant adjunctive option for the management of OM. The studies included in this review highlight its multifaceted biological properties, including antibacterial, antifungal, anti-inflammatory, wound-healing, and immunomodulatory activities [29]. The biological plausibility of these effects is supported by the strong association between the phenolic compounds present in propolis and its potent antioxidant capacity, which plays a critical role in neutralizing reactive oxygen species (ROS) generated during chemo- and radiotherapy [30,31]. In addition, the ability of propolis to inhibit the production of key pro-inflammatory cytokines, such as interleukin-1β (IL-1β), interleukin-6 (IL-6), and tumor necrosis factor alpha (TNF-α), directly targets the inflammatory phase of mucositis. Its antimicrobial activity further contributes to clinical benefit by reducing opportunistic infections, including oral candidiasis, through the control of pathogenic microorganisms such as Staphylococcus aureus and *Porphyromonas gingivalis*, thereby fostering a microenvironment conducive to mucosal healing and tissue regeneration [12,14,32].

When compared with conventional supportive therapies, propolis demonstrates several competitive advantages. Available evidence suggests that many traditional interventions primarily provide symptomatic relief, whereas propolis may exert more rapid anti-inflammatory effects and is associated with a relatively low incidence of adverse events [17,22]. Nevertheless, a critical appraisal of the literature indicates that clinical efficacy is not uniform and appears to be strongly dependent on the pharmaceutical formulation. A notable challenge in synthesizing the evidence on propolis lies in the inherent chemical heterogeneity derived from its botanical origin. While Brazilian Green Propolis (sourced from *Baccharis dracunculifolia*) is characterized by high concentrations of prenylated phenylpropanoids such as Artepillin C, European and Asian varieties (often sourced from *Populus* species or *Mangifera indica*) are predominantly rich in flavonoids (e.g., galangin, pinocembrin) and caffeic acid phenethyl ester (CAPE) [33,34]. These distinct chemical profiles may activate different biological pathways, complicating direct comparisons of efficacy. Furthermore, this review underscores a clear formulation-related dilemma: aqueous-based preparations and mucoadhesive gels were generally associated with favorable efficacy and tolerability profiles, whereas alcoholic extracts were poorly tolerated. Ethanolic extracts typically yield higher concentrations of bioactive non-polar compounds but may cause irritation or pain upon application to inflamed mucosa, whereas aqueous extracts, though better tolerated, may require higher concentrations to achieve comparable therapeutic effects. The pediatric study conducted by Tomaževič et al. [19] exemplifies the risks associated with inappropriate delivery vehicles, in which alcohol-based formulations caused intense burning sensations and vomiting, leading to poor adherence. Finally, the interpretation of clinical outcomes is frequently confounded by the use of multi-ingredient formulations; combinations with agents such as honey, aloe vera, or chamomile may exert synergistic anti-inflammatory or wound-healing effects, making it difficult to attribute observed benefits solely to propolis [16,17,23,24].

Despite its promising therapeutic profile, the strength of the current evidence base is substantially limited by methodological, pharmaceutical, and botanical heterogeneity. One of the most critical findings of this review is the frequent generic use of the term “propolis,” which obscures substantial variations in chemical composition driven by geographic origin and local flora. Distinguishing Brazilian green propolis from propolis derived from temperate or other regions is essential, as their chemical profiles and pharmacological activities differ markedly. The absence of detailed chemical characterization in most studies severely limits reproducibility and external validity. In addition, the internal validity of several trials is compromised by challenges in maintaining adequate blinding. The distinctive sensory properties of propolis, including color, taste, and odor, make the development of indistinguishable placebo formulations difficult, increasing the risk of performance and detection bias. Studies by Jen et al. [18] and Piredda et al. [25], for example, explicitly acknowledged compromised blinding, which may have led to overestimation of benefits, particularly for subjective outcomes such as pain intensity.

**Figure 2:**
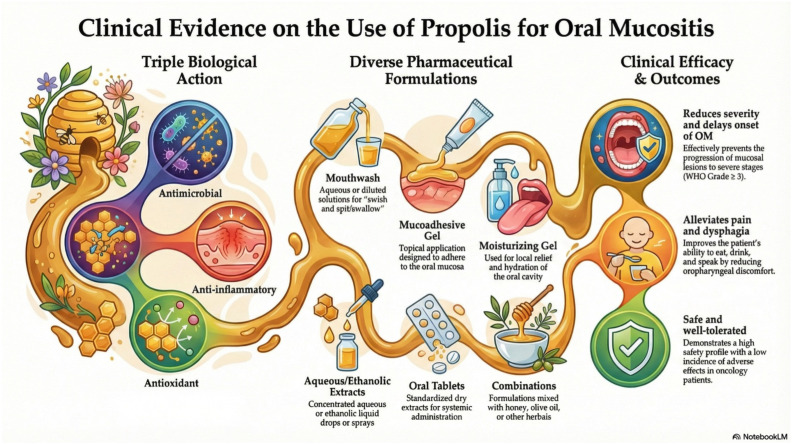
Schematic representation of the pharmaceutical formulations for propolis administration described in the studies included in this review, along with the corresponding clinical outcomes and identified limitations, emphasizing the need for methodological standardization and scientific validation. OM, oral mucositis. The figure was created using the NotebookLM tool. Google. (2026). NotebookLM (26 February 2026).

In summary, propolis can currently be categorized as an adjuvant agent with favorable preliminary evidence, given its broad biological activity, general acceptability, and low incidence of adverse effects. However, the available data remain insufficient to support a formal global recommendation for its use as monotherapy in the prevention or treatment of OM. Figure 2 summarizes the pharmaceutical formulations of propolis evaluated in the included studies, together with their main clinical outcomes and identified limitations. To advance the understanding of propolis in OM management, future research should prioritize the determination of optimal dosing regimens for different patient populations, including pediatric, adult, and immunocompromised individuals. Additionally, rigorous comparisons across standardized pharmaceutical formulations and well-designed, long-term randomized clinical trials are required to assess safety, efficacy, and potential adverse effects. Standardization of study designs and chemical characterization is fundamental to elucidate underlying mechanisms of action and to establish propolis as a reliable therapeutic option for OM associated with oncological treatments.

## 5. Final Considerations

In light of the ongoing need for robust evidence supporting the use of natural substances in the management of OM, the present review suggests that propolis may represent a safe adjuvant therapeutic option for the prevention and treatment of OM in patients undergoing radiotherapy, chemotherapy, or other antineoplastic interventions. However, the available evidence is characterized by methodological heterogeneity and, in some cases, mixed clinical outcomes, which preclude definitive conclusions regarding efficacy. Although certain studies have reported clinically relevant benefits such as pain relief, delayed onset of mucosal lesions, and reduced progression to more severe stages of OM, these findings should be interpreted with caution. At present, the clinical use of propolis should be individualized and considered experimental pending further well-designed, standardized clinical trials.

Further research is needed to strengthen the current evidence base. Future studies should prioritize: (1) well-designed randomized controlled clinical trials with greater methodological rigor; (2) standardization of propolis-based products regarding geographic origin, chemical composition, pharmaceutical formulation, concentration, dosage, and administration protocols; (3) additional laboratory investigations to more comprehensively elucidate the inflammatory and infectious mechanisms involved in the development and exacerbation of oral mucositis; and (4) stratification of clinical outcomes according to patient age, tumor location, and type of oncological treatment, thereby improving the clinical applicability, reproducibility, and reliability of the available evidence.

## Data Availability

No new data were created or analyzed in this study. Data sharing is not applicable to this article.
